# A multimodal approach combining Tele‐Neurorehabilitation and Bright Light Therapy for Dementia with Lewy Bodies patients: a pilot study

**DOI:** 10.1002/alz.095102

**Published:** 2025-01-09

**Authors:** Lucia Guidi, Luisa Sambati, Davide Braghittoni, Elettra Capogna, Greta Venturi, Annalisa Bosco, Chiara La Morgia, Giulia Amore, Raffaele Lodi, Caterina Tonon, Micaela Mitolo

**Affiliations:** ^1^ IRCCS Istituto delle Scienze Neurologiche di Bologna, Bologna, Bologna Italy; ^2^ University of Bologna, Bologna, Bologna Italy; ^3^ University of Catania, Catania, Catania Italy; ^4^ IRCCS Istituto delle Scienze Neurologiche di Bologna, Bologna Italy; ^5^ University of Bologna, Bologna Italy; ^6^ IRCCS Istituto delle Scienze Neurologiche di Bologna, Bologna, Italy Italy; ^7^ University of Parma, Parma Italy

## Abstract

**Background:**

Current evidence on non‐pharmacological treatments in Dementia with Lewy Bodies (DLB) are relatively few and limited by small sample sizes. The goal of this pilot study was to test the efficacy of a new multimodal treatment that combines Tele‐Neurorehabilitation and “Bright Light” Therapy (BLT) in a sample of DLB patients.

**Method:**

Eighteen DLB patients (7F; 74.64 ± 7.11 years, mean age ± SD; education 12.44 ± 3.64 years) and eighteen age‐matched controls (only for baseline comparison) (10F; age 69.28 ± 9.88 years; education 12.94 ± 4.92 years) underwent an extensive neuropsychological battery and sleep quality assessments with self‐report questionnaires and wrist actigraphy. DLB patients were randomly assigned to the active group that received Tele‐Neurorehabilitation plus BLT, or to the control group, that received only Tele‐Neurorehabilitation (plus Sham BLT). An *ad hoc* protocol named S.M.A.R.R.T (Spatial abilities, Memory, Attention and executive functions, Reasoning, Reaction Times) including 15 rehabilitation sessions was implemented into a virtual platform; BLT was administrated through specific glasses enriched with blue light at 468 nm. Statistical analysis was conducted using ANCOVA for normal data distribution and Mann‐Whitney U test for non‐normal values (Bonferroni correction at p<0.05).

**Result:**

DLB patients at baseline had worse performance in different cognitive domains (i.e. visuo‐spatial and visuo‐perceptual domains, and semantic fluency) and worse sleep quality than controls (p<0.05). At baseline the two DLB subgroups (active and sham) showed no differences in neuropsychological scores, nor in sleep measures. After correction for multiple comparisons, although not statistically significant, the active group showed trend improvements in sleep measures (i.e. Epworth Sleepiness Scale baseline score = 7.4; post‐treatment score = 7.2) and a reduction in neuropsychiatric symptoms (i.e. Neuropsychiatric Inventory score = 13.6; post‐treatment score = 12.3), whereas sham group showed the opposite trend.

**Conclusion:**

These preliminary results show beneficial effects of a multimodal approach that combines Tele‐Neurorehabilitation and BLT, highlighting BLT as a promising treatment to improve sleep disturbances and neuropsychiatric symptoms in DLB patients. Further studies with bigger samples are needed to confirm these results and to corroborate its clinical application.

**Funding:**

This study has been supported by the Italian Ministry of Health (#GR‐2019‐12369242).

